# Selective Impact of Disease on Coral Communities: Outbreak of White Syndrome Causes Significant Total Mortality of *Acropora* Plate Corals

**DOI:** 10.1371/journal.pone.0132528

**Published:** 2015-07-06

**Authors:** Jean-Paul A. Hobbs, Ashley J. Frisch, Stephen J. Newman, Corey B. Wakefield

**Affiliations:** 1 Department of Environment and Agriculture, Curtin University, Perth, Australia; 2 ARC Centre of Excellence for Coral Reef Studies, James Cook University, Townsville, Australia; 3 Western Australian Fisheries and Marine Research Laboratories, Department of Fisheries, Government of Western Australia, North Beach, Australia; Università di Genova, ITALY

## Abstract

Coral diseases represent a significant and increasing threat to coral reefs. Among the most destructive diseases is White Syndrome (WS), which is increasing in distribution and prevalence throughout the Indo-Pacific. The aim of this study was to determine taxonomic and spatial patterns in mortality rates of corals following the 2008 outbreak of WS at Christmas Island in the eastern Indian Ocean. WS mainly affected *Acropora* plate corals and caused total mortality of 36% of colonies across all surveyed sites and depths. Total mortality varied between sites but was generally much greater in the shallows (0–96% of colonies at 5 m depth) compared to deeper waters (0–30% of colonies at 20 m depth). Site-specific mortality rates were a reflection of the proportion of corals affected by WS at each site during the initial outbreak and were predicted by the initial cover of live *Acropora* plate cover. The WS outbreak had a selective impact on the coral community. Following the outbreak, live *Acropora* plate coral cover at 5 m depth decreased significantly from 7.0 to 0.8%, while the cover of other coral taxa remained unchanged. Observations five years after the initial outbreak revealed that total *Acropora* plate cover remained low and confirmed that corals that lost all their tissue due to WS did not recover. These results demonstrate that WS represents a significant and selective form of coral mortality and highlights the serious threat WS poses to coral reefs in the Indo-Pacific.

## Introduction

Coral disease affects more than 130 species [1–3] and some taxa appear to be particularly susceptible to infection and mortality (e.g. Acroporids and Pocilloporids: [2]). These taxonomic differences in disease susceptibility can lead to long-lasting shifts in community composition (e.g. [4]). Disease prevalence and mortality in corals has also been found to vary spatially across regions [2], sites [5,6], and depths [7], and may indicate that some habitats (e.g. shallow and offshore reefs [2,7]) are more vulnerable than others.

Coral diseases are a significant and increasing threat to coral reefs worldwide [2,8]. They have already heavily impacted Caribbean reefs [4,8,9], and are increasing at rapid rates on Pacific Ocean reefs [2]. While a broad range of coral diseases and syndromes have been reported [1], there is a need to understand the effects of each disease on coral communities. To identify the most susceptible taxa and most vulnerable habitats, it is necessary to undertake spatial assessments of taxon specific patterns of disease and mortality. Quantifying diseased-induced mortality has been identified as a research priority in order to assess the impact of diseases relative to other causes of coral mortality [2].

White diseases and white syndrome (WS) are the most widespread and destructive of the coral diseases that impact coral reefs. These diseases have caused coral mortality throughout the Caribbean and Pacific Oceans [2,4–6,8–14]. While white diseases have heavily impacted reefs in the Caribbean [4], the distribution and prevalence of WS is increasing rapidly in the Pacific Ocean [2]. The impact of WS on coral reefs in the Indian Ocean is unknown.

WS can affect a range of coral taxa [1,2,10], but acroporid species appear most susceptible during initial outbreaks [2,11,13]. WS prevalence can vary between reefs and sites, and is most commonly reported from shallow waters (3 to 14 m), although research is required to determine if prevalence varies with depth [2,5,6]. WS outbreaks have been linked to elevated water temperatures, high cover of all live corals [13], high cover of host corals [15] and the prevalence of certain *Vibrio* species [16,17]. Discerning the role of different characteristics of the coral community (e.g. cover of all live corals, cover of host corals, number of host corals) is important to understanding disease dynamics and density dependent effects.

Previous studies of WS typically report patterns of prevalence and rates of disease progression or tissue loss during the initial outbreak [2,12–14]. However, these measures may not be reliable predictors of WS-induced coral mortality because they provide snap-shots that may under or over-estimate the total mortality following a disease outbreak. For example, if surveys are conducted in the preliminary stages of an outbreak, then the total mortality of colonies and total number of species affected may be underestimated [1,9,18]. In contrast, if corals develop disease resistance or partially recover from initial tissue loss [5] then one-off surveys may overestimate mortality. Therefore, to gain a comprehensive understanding of the impact of WS on coral reefs, surveys optimally need to be conducted during and immediately after an outbreak to determine the proportion of affected colonies and rates of total mortality, respectively.

In 2008 there was a significant outbreak of WS at Christmas Island in the eastern Indian Ocean. Spatial and taxonomic patterns in WS prevalence during this initial outbreak were documented in a previous study [19]. WS was taxonomically selective with *Acropora* plate corals representing 97% of affected corals [19]. Of all the surveyed *Acropora* plate corals, 13% were affected by WS [19], which is a higher prevalence than other locations [2,5]. Like other WS field studies, the previous study at Christmas Island did not investigate the fate of the WS-affected colonies. The overall aim of this study was to determine the ultimate outcome of the WS outbreak at Christmas Island by quantifying WS-induced mortality rates and assessing the impact on the coral community. Given that *Acropora* plate corals were the taxa most susceptible during the initial WS outbreak, we focused on mortality patterns in this taxon. Specifically, the questions addressed in this study were: 1. Was there a decline in the mean number of *Acropora* plate corals following the WS outbreak? 2. Did mortality in *Acropora* plate corals vary between sites and depths? 3. How did WS-induced mortality impact on live hard coral cover? 4. Were spatial patterns in WS-induced mortality related to: the number of live *Acropora* plates; live *Acropora* plate cover; or live hard coral cover? 5. Were mortality rates in *Acropora* plate corals linked to the proportion of colonies affected by WS during the outbreak?

## Materials and Methods

### Study site

Christmas Island (10°30’S, 105°40’E) is a single oceanic island that rises abruptly from the surrounding seafloor (> 4000 m deep) and is located 350 km southwest of Java, Indonesia. Its 139 km coastline is fringed by a narrow coral reef (20–100 m wide) before dropping precipitously towards the seafloor. Field surveys were conducted along this reef at eight sites on the western (2 sites), northern (4 sites) and eastern (2 sites) coastlines of the island (see [19, 20] for site details). The field surveys for this study were conducted in accordance with permits issued by Parks Australia and Department of Fisheries, Government of Western Australia

### WS outbreak

WS was first observed at Christmas Island in February 2008. A full description of the WS lesions and the spatial and taxonomic characteristics of the outbreak are provided by Hobbs & Frisch [19]. Even though 172 species of scleractinian corals have been recorded at Christmas Island [20], WS was predominately restricted to the three *Acropora* plate species: *A*. *clathrata*, *A*. *cytherea* and *A*. *hyacinthus*. Due to this taxonomic bias in WS susceptibility, this study focussed on *Acropora* plate corals.

The WS outbreak of 2008 was most severe during February, March and April [19], after which the prevalence of WS decreased. By September, no new cases of WS were seen. Although 3% of plate corals still had signs of WS in September, the WS front was proceeding through the last of the live tissue. By late October, there were no signs of active WS on any *Acropora* plate corals.

### Survey design

Two different surveys were used to document WS induced mortality in *Acropora* plate corals. The first surveyed measured changes in the number of live *Acropora* plate corals during and after the WS outbreak and the second survey measured the number of recently-killed *Acropora* plate corals following the WS outbreak. The first survey was a repeat of the same methods used during the initial outbreak [19], while the second survey represents a tailored increase in survey effort that was designed to provide greater detail on spatial variability and a more direct estimate of the WS-induced mortality.

The purpose of the first survey was to determine whether the density of *Acropora* plate corals had decreased following the observed WS outbreak. To do this, eight of the ten sites surveyed during the initial outbreak (described in [19, 20]) were resurveyed in October 2008. The two south coast sites could not be resurveyed due to adverse weather conditions. This survey counted the number of live *Acropora* plate corals in three replicate 30 m by 5 m transects at the 5 m depth. During the initial outbreak, four of the ten sites were also surveyed at 20 m depth [19], and these four sites were also resurveyed (in October) at 20 m depth using the same transects (three replicate 30 m by 5 m).

**Fig 1:**
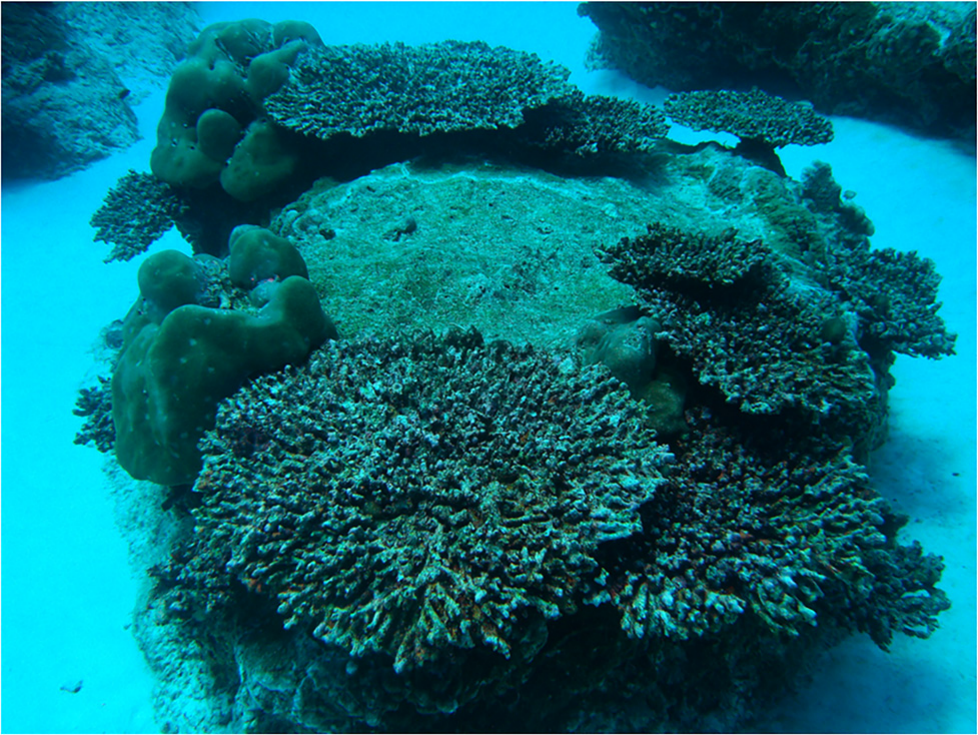
Appearance of *Acropora* plate corals approximately 8 months after the White Syndrome (WS) outbreak and representation of the change in coral community composition caused by the significant and selective WS mortality. The photograph was taken during the follow-up surveys in October 2008.

To provide a more direct measure of mortality rates in *Acropora* plate corals specifically and to allow for comparisons between depths (5 and 20 m) across all eight sites, an additional survey was conducted at both depths at the eight monitoring sites [19]. In this second survey, the number of dead *Acropora* plate colonies was recorded within three replicate 30 m by 10 m transects (at each depth and site). The mortality rate was then calculated as the number of dead *Acropora* plate coral colonies divided by the total number of live and dead *Acropora* plate coral colonies. We used 42 tagged *Acropora* plate corals that had been affected by WS and subsequently died as references for the visual appearance of corals that had died due to the WS outbreak (Fig 1). Corals that recently died due to WS had intact fine-scale morphological structures and were colonised by green and brown turfing algae and could be easily distinguished from corals that had died in previous years (which had lost fine-scale morphological features and were colonised by other organisms such as coralline algae and coral recruits). Furthermore, during the initial WS outbreak (February to April), previously dead colonies accounted for less than 0.3% of all plate corals at the survey sites, and thus the number of dead plate corals in the follow-up survey (October) was strongly indicative of recent WS induced mortality. Between the initial outbreak and the follow-up survey (approximately 6 months later) no other obvious environmental impacts were observed (e.g. coral bleaching, storms, floods, *Acanthaster* outbreaks). Thus we have no reason to believe that anything other than WS caused the observed mortality in *Acropora* plate corals. Furthermore, we observed WS progress and kill 40 tagged plate corals during late April and May, providing further support that dead plate corals observed in October had died due to WS.

To examine how WS progressed through *Acropora* corals, underwater photographs and repeated observations were made of WS-affected colonies chosen haphazardly at Flyingfish Cove. These corals were tagged and repeated observations (on two to eight occasions) revealed that following the initial onset of WS, most *Acropora* plate corals had no tissue left within 1–3 months (i.e. they suffered total mortality). However, two of the 42 plate corals affected by WS were still alive after this time and although they had lost some tissue (i.e. partial mortality), the characteristic white band of WS was no longer visible. Thus, colonies that exhibited partial mortality (approximately 5%) during our follow-up field surveys were excluded from analyses because it was not known whether they would survive into the future. Therefore, our analyses focused on colonies that were either completely alive or completely dead.

To determine whether mortality was linked to live coral cover or *Acropora* plate coral cover, these variables were quantified using six replicate 2 m by 2 m quadrats per depth at each of the 8 sites. Each quadrat was placed randomly (using the numbered measurements on the transect tape) within the transects described above and within each quadrat, percent cover was visually estimated for each of the following categories: live hard coral (Scleractinia), soft coral, crustose coralline algae, epithelial algal matrix, sand, rubble, rock-bare substrate and “other” (sponges, zoanthids, gorgonians, anemones and macro-algae). The live hard coral category was further divided into 8 morphological categories: branching, corymbose, plate (i.e. tabular *Acropora*), foliose, massive, columnar, encrusting and free-living (i.e. fungids) following Veron [21]. The quadrat approach was used during the initial outbreak, and repeated at the same sites (but using different random positions on the transects) after the outbreak (October).

### Statistical analyses

To determine the severity and spatial impact of WS, the mean number of *Acropora* plates were compared between survey periods and sites using a two-way analysis of variance (ANOVA) following log(*x*+1) transformation. To examine patterns of WS-induced mortality, the proportion of dead *Acropora* plate corals were compared between sites and depths with a two-way ANOVA following arcsine transformation. Changes in percent cover of hard coral categories between survey periods were examined using t-tests on arcsine transformed data. Only coral categories that had a mean coverage of more than 3% of the benthos were analysed. Linear regressions were used to examine whether the proportion of dead *Acropora* corals at survey sites post-outbreak was related to the following measures recorded during the initial outbreak: mean number of live *Acropora* plate corals, percent live *Acropora* plate cover, percent live hard coral cover, and percent of WS-affected *Acropora* plate corals.

## Results

**Fig 2:**
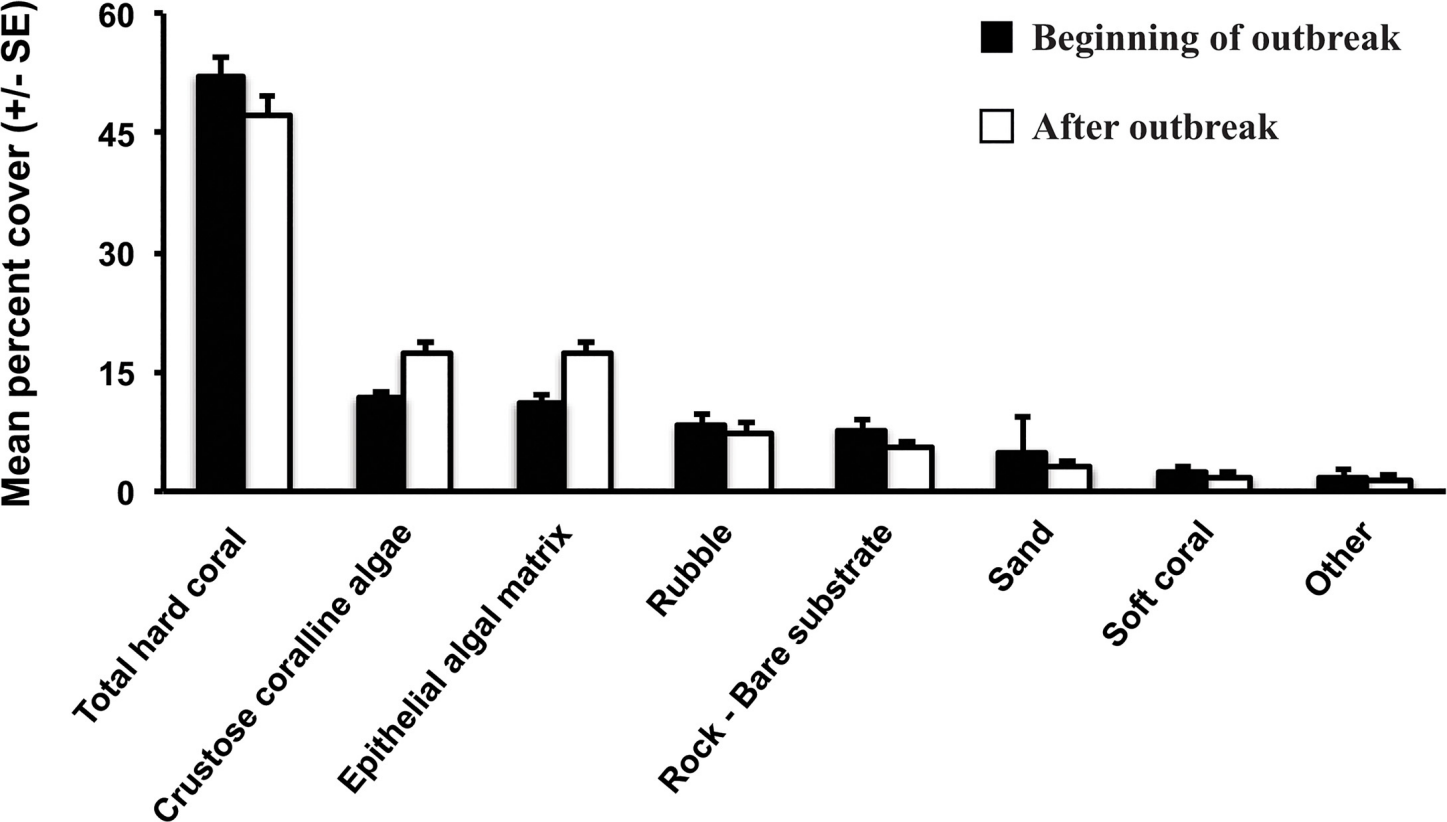
Mean percent (± SE) cover of the most common habitat categories at the beginning of (February to April 2008—black bars), and after (October 2008—white bars), the White Syndrome outbreak at Christmas Island. The category “Other” includes sponges, anemones, *Halimeda*, fleshy macroalgae, gorgonians and zoanthids.

At the beginning of the WS outbreak (February to April, 2008) mean live hard coral cover was 52.1% (±2.4 SE) on the reefs around Christmas Island. Following the outbreak, mean live hard coral cover decreased to 47.4% (±2.3 SE), while the mean of cover of crustose coralline algae increased from 11.7% (±0.9 SE) to 17.3% (±1.4 SE) and epithelial algal matrix increased from 11.1% (±1.0 SE), to 17.2% (±1.7 SE) (Fig 2). Other common habitat categories included rubble (8.2% ±1.5 SE), rock-bare substrate (7.7% ±1.2 SE) and soft coral (1.7% ±1.6 SE)(Fig 2).

**Fig 3:**
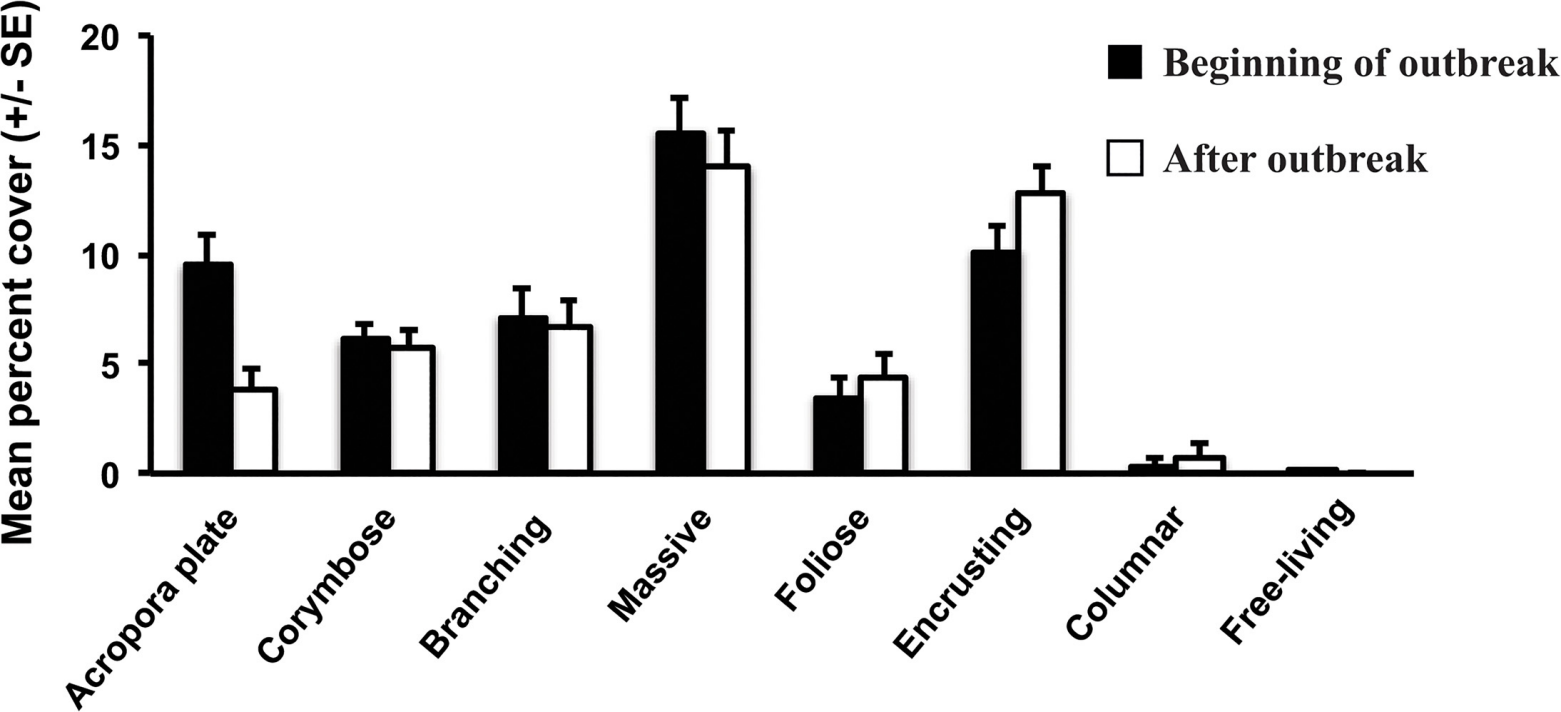
Mean percent (± SE) cover of the six most common coral morphologies at the beginning of (February to April 2008—black bars), and after (October 2008—white bars), the White Syndrome outbreak at Christmas Island.

**Fig 4:**
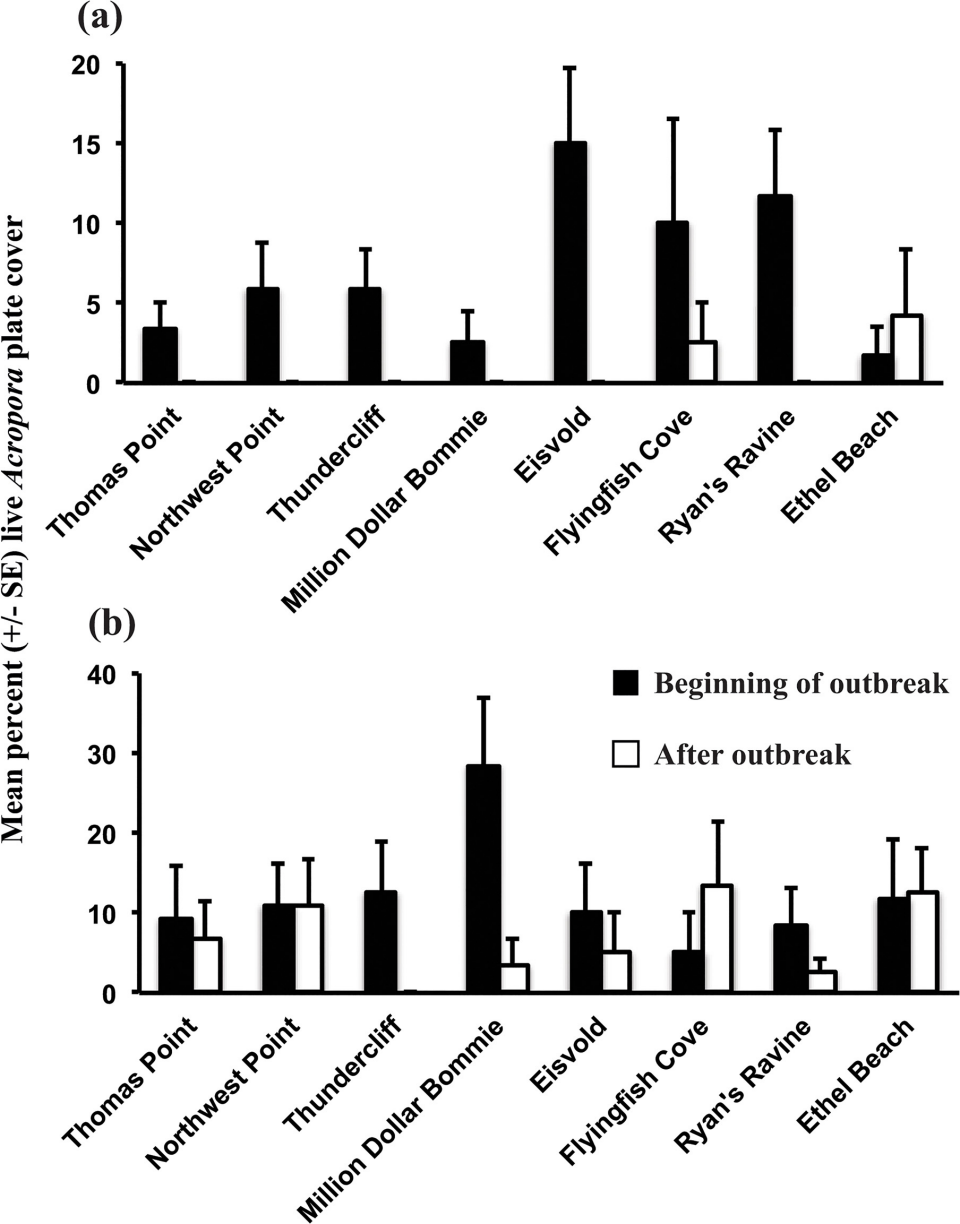
Mean percent (± SE) *Acropora* plate cover at eight sites at (a) 5 and (b) 20 m depth at the beginning of (February to April 2008—black bars), and after (October 2008—white bars), the White Syndrome outbreak at Christmas Island.

**Fig 5:**
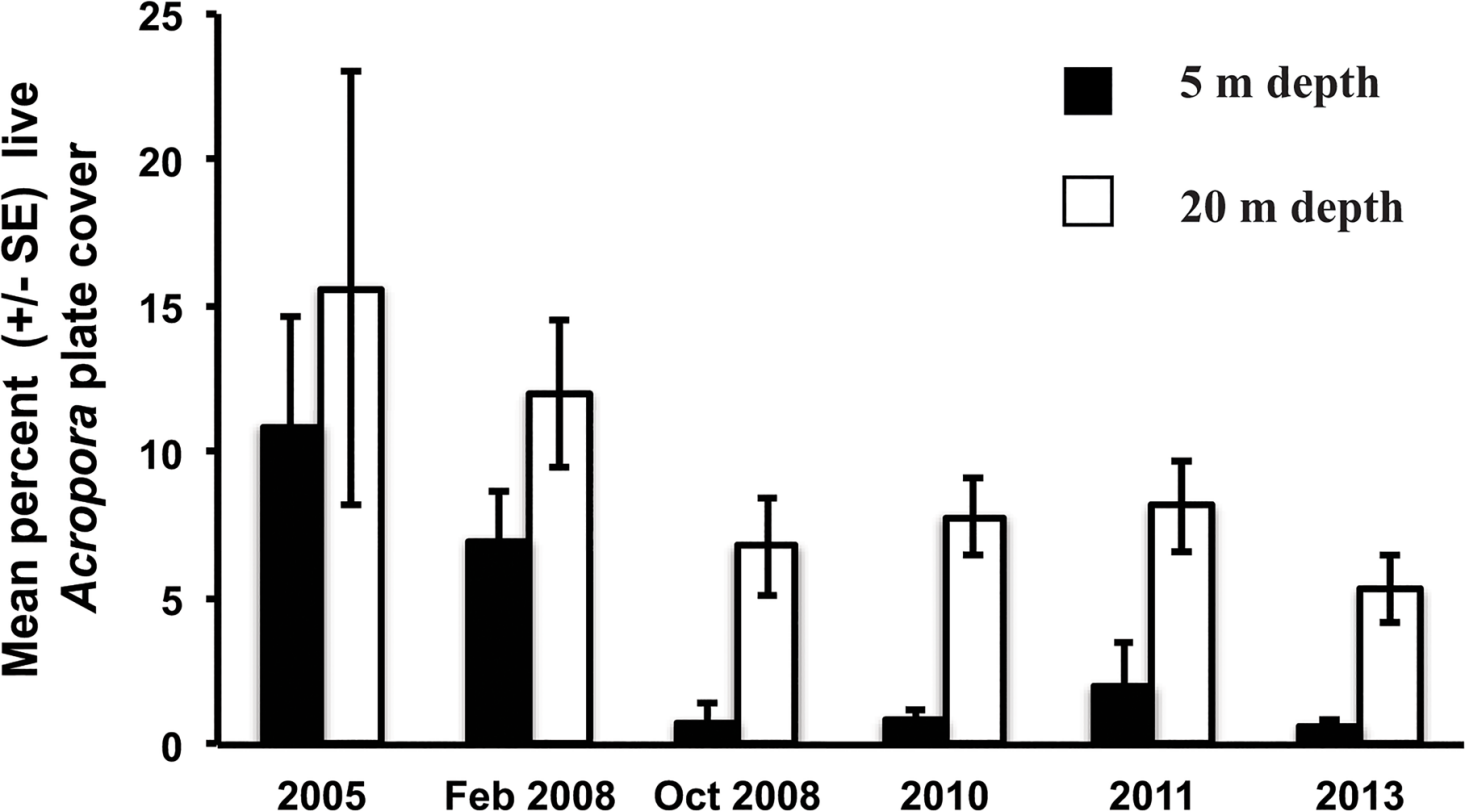
Mean percent live coral cover (± SE) of *Acropora* plate corals from 2005 to 2013. February 2008 represents the beginning of the White Syndrome outbreak and October 2008 represents the end of the outbreak. Values for 2005 are from [27], and are based on seven of the eight study sites surveyed from 2008 to 2013. Values are presented for 5 m (black bars) and 20 m (white bars) depths.

The outbreak of WS predominately affected *Acropora* plate corals (Fig 3). Following the outbreak of WS, mean *Acropora* plate cover (across all sites and depths) declined significantly from 9.5 (±1.6 SE) to 3.8% (±1.2 SE)(*t*-test: *t* = 3.45, d.f. = 14, *p* = 0.003), while the other categories of hard corals (corymbose, branching, massive, foliose, encrusting) experienced minimal change in percent cover or were too rare to be analysed (<3% coverage: columnar and free-living)(Fig 3). A closer examination revealed that the majority of the decline in the cover of *Acropora* plate corals occurred in the shallows. Mean *Acropora* plate cover declined from 7.0 (±1.7 SE) to 0.8% (±0.6 SE) at 5 m depth following the WS outbreak (*t*-test: *t* = 3.45, d.f. = 14, *p* = 0.004, n = 8, Fig 4A). Whereas at the 20 m depth, the decline in mean *Acropora* plate cover (from 12.0 ± 2.5 SE to 6.8% ±1.7 SE, Fig 4B) was less severe and not statistically significant (*t*-test: *t* = 1.71, d.f. = 14, *p* = 0.11, n = 4, Fig 5). Mean percent cover of *Acropora* plate coral has remained low five years after the 2008 WS outbreak (Fig 5).

**Fig 6:**
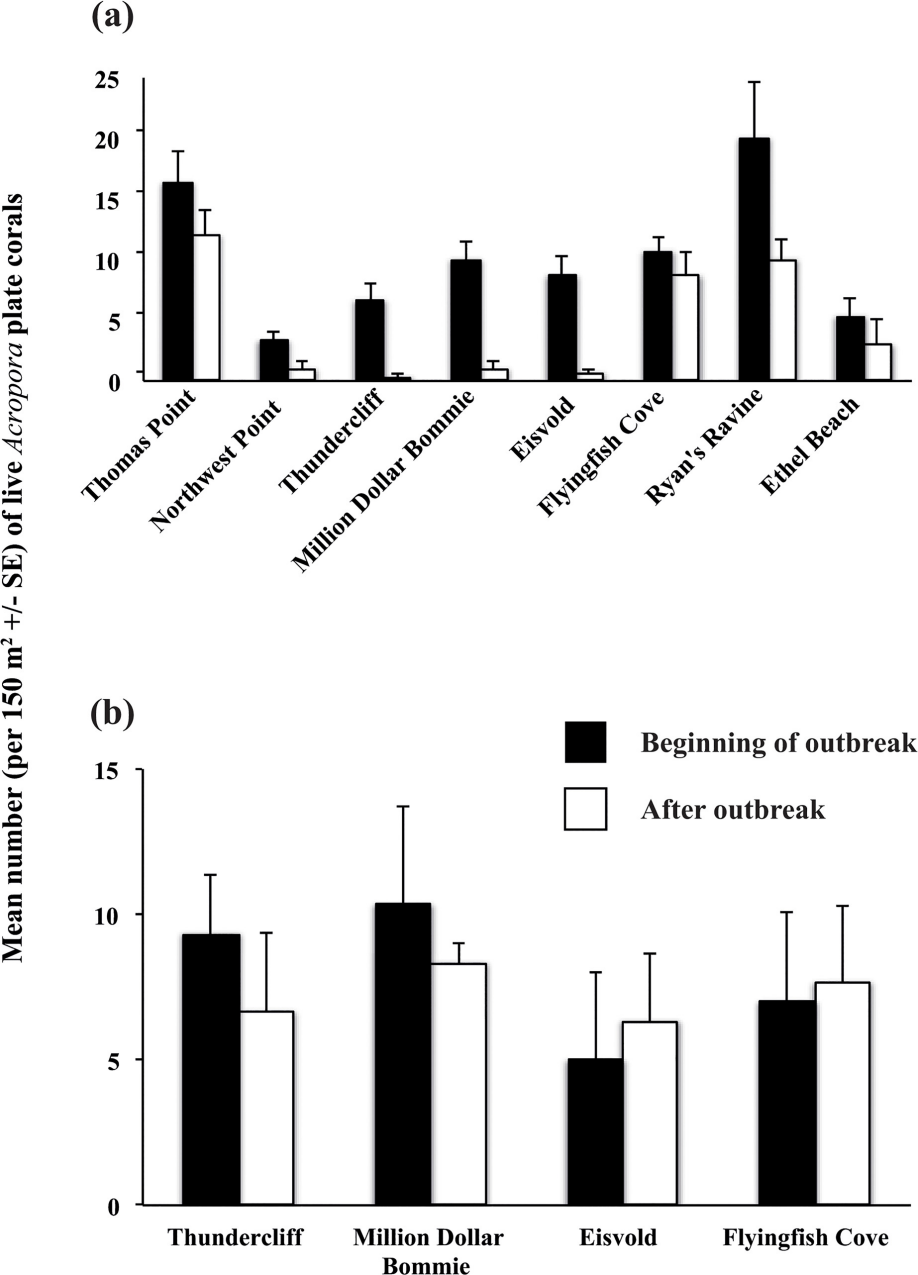
Mean number of live *Acropora* plate corals (colonies per 150 m^2^ ± SE) during (black bars), and 6 to 8 months after (white bars), the outbreak of White Syndrome at Christmas Island. Data were recorded at (a) 5 m depth at eight sites, and (b) 20 m depth at four sites.

Following the outbreak of WS at Christmas Island, there was a general decline in the mean number of live *Acropora* plate corals in the shallows (5 m). However, the magnitude of decline varied considerably between sites, ranging from 28 to 96% (two-way ANOVA interaction: *F*_1,7_ = 4.3, *p* = 0.002, n = 8, Fig 6A). At 20 m depth, there was no significant change (*p* > 0.4, n = 4) in the mean number of live *Acropora* plate corals across sites (Fig 6B).

**Fig 7:**
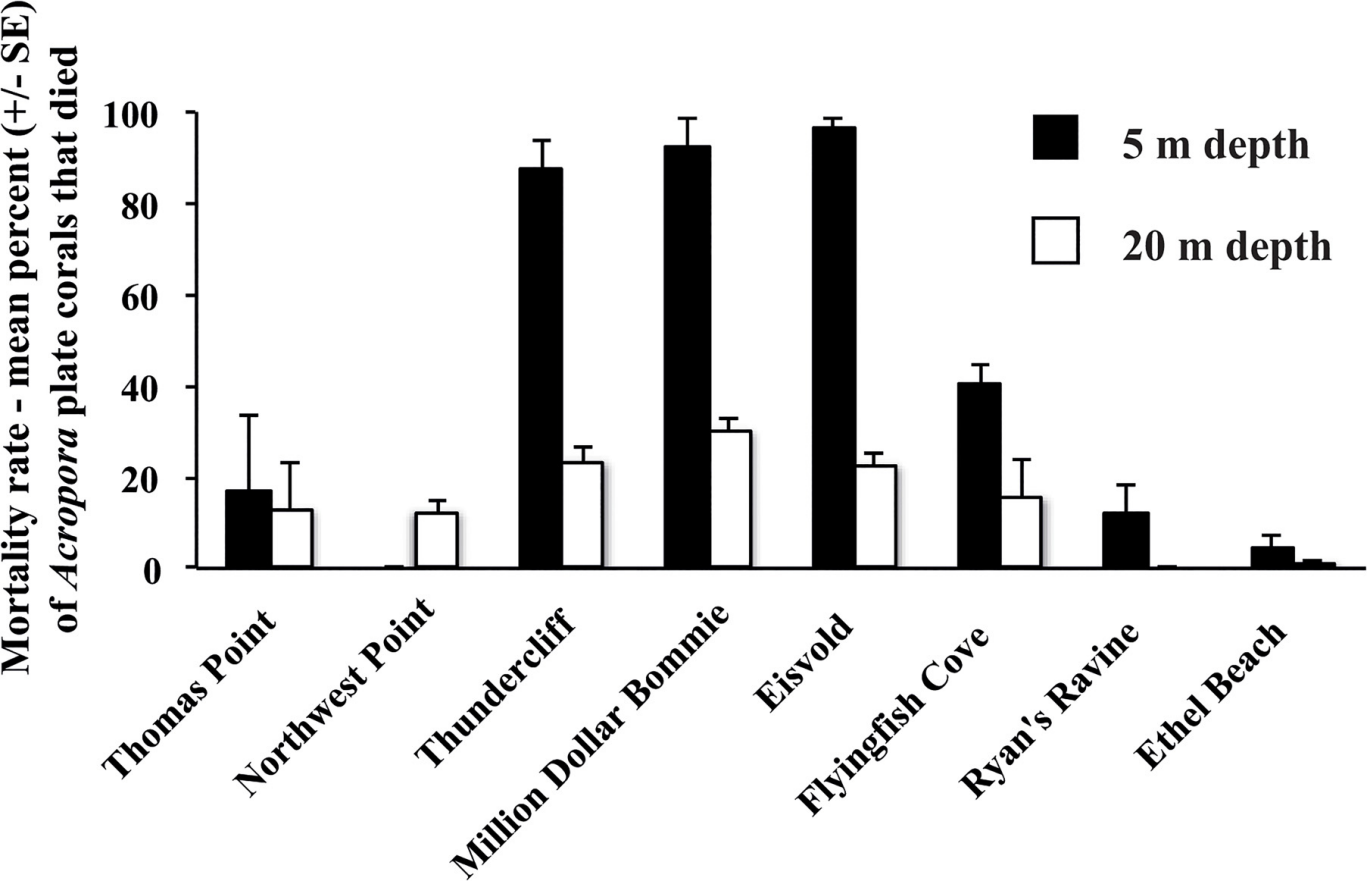
Mean mortality rate (% ± SE) of *Acropora* plate corals as measured by the percent of recently dead colonies recorded in surveys 6 to 8 months after the White Syndrome outbreak began at Christmas Island. Data were recorded at 5 m (black bars) and 20 m depths (white bars) at eight survey sites in October 2008.

Site and depth specific responses were also evident in the proportion of *Acropora* plate corals that died following the WS outbreak (two-way ANOVA interaction: *F*_1,7_ = 13.2, *p* < 0.0001, Fig 7). Across all surveyed sites and depths, 36% of *Acropora* plate corals died (353 of 973 colonies). Mortality rates in the shallows (5 m) ranged from 0 to 96% per site and were generally higher than those recorded at 20 m depth (0 to 30%). At one site (Northwest Point), however, the mortality rate was higher at 20 m depth (12%) than at 5 m depth (0%). This contrasting result could represent a sampling artifact related to the rarity of *Acropora* plates remaining in the shallows at Northwest Point. The mean number of live *Acropora* plate corals at 5 m depth at this site did decline following the WS outbreak (Fig 6A).

**Fig 8:**
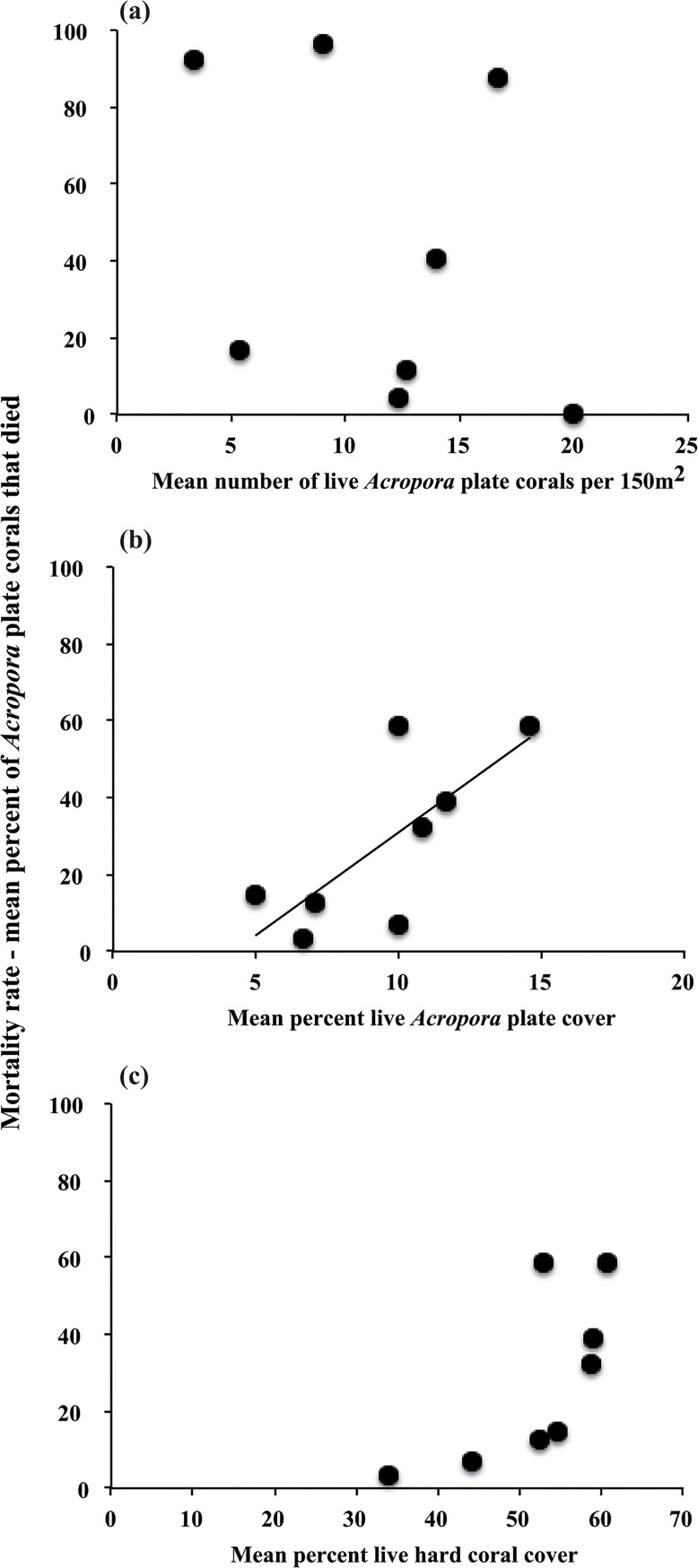
The relationships between the mortality rate as measured by the percent of recently dead *Acropora* plate corals following the White Syndrome (WS) outbreak and the (a) initial mean number of live *Acropora* plate colonies, (b) initial mean percent live *Acropora* plate cover, and (c) initial mean percent total live hard coral cover at eight sites around Christmas Island. The initial data was recorded during the outbreak of WS (February to April 2008), before total mortality of plate corals. Data for (a) are from 5 m depth only because equivalent data was not collected at all sites at 20 m depth, and data for (b) and (c) are from both depths combined.

**Fig 9:**
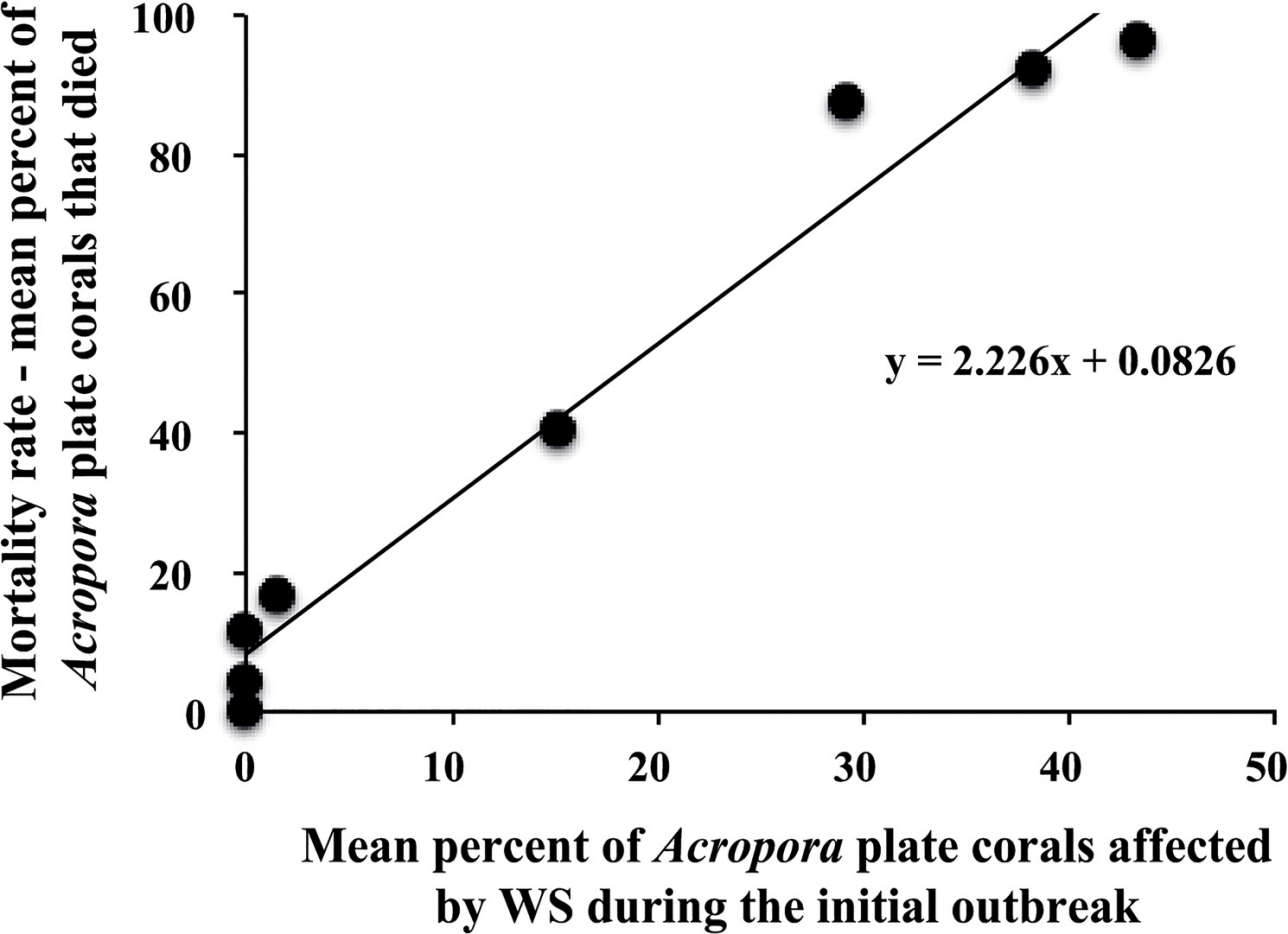
The relationship between the mean percent of *Acropora* plate corals affected by White Syndrome during the initial outbreak and the mortality rate as measured by mean percent of dead *Acropora* plate corals recorded 6 to 8 months after the outbreak. Data are from surveys at 5 m depth at eight sites around Christmas Island.

Regression analysis revealed that differences in site-specific mortality rates of *Acropora* plate corals was not correlated with the initial number of *Acropora* corals (*F* = 0.13, *p* = 0.74, *r* ^2^ = 0.02, Fig 8A) but was positively related to the initial percent cover of live *Acropora* plate coral (*F* = 7.21, *p* = 0.036, *r* ^2^ = 0.55, Fig 8B). Mortality rates were weakly associated with total live hard coral cover (*F* = 5.45, *p* = 0.058, *r* ^2^ = 0.48, Fig 8C). The proportion of WS-affected *Acropora* plate corals recorded at a site during the WS outbreak was a reliable predictor of the subsequent level of *Acropora* plate coral mortality at that site (*F* = 175.71, *p* < 0.0001, *r* ^2^ = 0.97, Fig 9).

## Discussion

Preceding studies examining the impact of WS on Indo-Pacific coral reefs have focused on the proportion of live corals affected by WS and disease progression to predict potential effects [2,12–14]. This study builds on this existing knowledge by determining the ultimate fate of corals affected by WS and thereby characterising how WS impacts on a coral reef community in the Indo-Pacific. WS caused total mortality in up to 96% of *Acropora* plate corals at some sites and depths. Mortality rates varied between sites and were generally greater at 5 m depth than at 20 m depth. Mortality rates were primarily related to the proportion of corals affected by WS during the outbreak and the percent of live *Acropora* cover during the outbreak. Following the WS outbreak, there was a considerable decline in *Acropora* plate coral cover, but no change in the cover of other hard corals surveyed. It is clear that WS has changed the coral community at Christmas Island through significant and selective mortality of *Acropora* plate corals.

There were noticeable spatial differences in mortality rates of *Acropora* plate corals affected by WS. Differences between sites in the mortality rates (28 to 96%) indicate that site specific differences in the conditions promoting WS. In response to thermal stress, coral bleaching varies between sites due to spatial differences in environmental conditions, such as hydrodynamics and productivity [22,23]. These variations in environmental conditions may cause spatial differences in susceptibility of corals to WS (including the contribution of past stressors to current disease susceptibility [24]), or may increase the abundance of pathogens linked to WS outbreaks (e.g. [16]) or the spread of WS. Spatial differences in the conditions that may facilitate transmission of WS between corals (e.g. host density – see below) could also explain differences among sites. Higher disease prevalence in shallow waters was observed in this study, and has been reported previously (e.g. [7,25]). This may be due to greater fluctuations in environmental conditions in shallow waters [25], or differences in the amount of light penetration [26] or differences in host densities or coral communities between depths.

*Acropora* plate corals represented 97% of colonies affected by WS during the outbreak at Christmas Island [19] and we saw no evidence that WS switched host during or after the outbreak. WS represents a significant threat to populations of *Acropora* plate corals at Christmas Island given that it almost killed all of these corals at some sites and depths. Furthermore, due to the positive relationship between initial percent cover of live *Acropora* plate coral and subsequent WS-induced mortality rates, the sites that had the highest cover of *Acropora* plate corals during the outbreak also had the highest mortality rates. WS caused a two and half fold decrease in *Acropora* plate coral cover across the study sites, including an eight fold decrease in the shallows. Acroporid corals were the greatest contributor to coral cover in 2005 (13.4% cover across all sites and depths [27]), however, after the 2008 outbreak, *Acropora* plate cover at 5 m depth declined from 7.0 to 0.8% and 5 years later this cover has remained low (2013 = 0.7%). There is no relationship between coral cover and coral species richness at Christmas Island [28], and other Indo-Pacific reefs [29,30], and thus the decrease in three (*A*. *clathrata*, *A*. *cytherea* and *A*. *hyacinthus*) of the 172 species at Christmas Island [20] has a disproportional effect on the coral community because these *Acropora* plate corals were the group contributing the highest cover of live coral before the disease outbreak [19, 27].

Taxonomic variations in the vulnerability of corals to disturbances are often attributed to differences in physiology (e.g. faster growth and respiration in *Acropora*) and morphology [31–34]. As found in this Indian Ocean study, acroporid corals are also the taxa most susceptible to WS in the Pacific Ocean [2,13]. Therefore WS is likely to cause predictable changes in the community composition of Indo-Pacific reefs (i.e. a decrease in *Acropora*), as has occurred in the Caribbean following outbreaks of white band disease [4]. Furthermore, the loss of *Acropora* corals due to WS may compound the effects of other disturbances in the region [35] because *Acropora* corals are also the most susceptible to other impacts (e.g. coral bleaching and crown-of-thorns starfish [36,37]).

The distribution and prevalence of WS is increasing throughout the Indo-Pacific, with some reefs experiencing a 150 fold increase in prevalence within 5 years [2]. While little previous research has been undertaken to determine the levels of mortality at sites affected by WS, the high mortality rates documented in this study, combined with predicted increases in WS prevalence, establishes that WS poses a significant threat to coral reefs. Further studies are needed to document mortality rates of WS and other coral diseases to determine their importance relative to other sources of mortality (e.g. bleaching, crown-of-thorns starfish), and ultimately to determine what triggers disease outbreaks. Such studies would also reveal whether corals can develop immunity to specific diseases. That some *Acropora* plate corals at Christmas Island were not affected by WS (despite being close to affected colonies) suggests some degree of intraspecific variation in disease susceptibility, which may contribute to WS immuno-resistance in the future.

In this study, site-specific differences in total mortality were a reflection of site-specific differences in the proportion of plate corals affected by WS during the initial outbreak. This strong positive relationship between affected colonies and mortality rates indicates that there was no spatial variability in the ability for *Acropora* plate corals to recover once affected by WS. Furthermore, the slope of the affected-mortality relationship was 2.2 (Fig 9), which means the number of corals that died from WS was more than double the number of WS-affected plate corals recorded during the initial surveys. The initial surveys occurred at the beginning of the outbreak and the additional mortality represents colonies that became affected by WS after our initial surveys. Indeed, we observed numerous new cases of WS in Flyingfish Cove in the months (March to August) after our initial surveys (February) at this site. That outbreaks continue for several months highlights the need for extended monitoring studies to document the full impact of coral disease.

Some studies report that coral can regrow tissue and recover following WS-induced tissue loss (e.g. [5,38]), however most WS-affected plate corals in this study experienced total loss of tissue with no signs of recovery. Five years after the initial WS outbreak and the remains of the 40 tagged corals that lost all their tissue during the outbreak have not regrown any tissue but rather have been colonized by a range of organisms including coral recruits. The same was observed for dead plate corals at the other survey sites and confirms that the WS outbreak at Christmas Island caused total mortality of *Acropora* plate corals. However, 5% of WS-affected plate corals experienced partial mortality, and although these corals were not monitored, it cannot be discounted that some tissue regrowth could have occurred in these colonies.

Since spatial patterns in mortality reflect spatial patterns in WS-affected corals during the outbreak, future research should focus on spatial and temporal patterns in how corals are affected to elucidate the cause(s) of WS outbreaks. At Christmas Island, the proportion of *Acropora* corals affected by the WS outbreak was positively associated with both *Acropora* plate coral cover and the number of host colonies (and not live hard coral cover [19]), indicating high host density plays a role in WS prevalence, possibly through overcrowding or transmission facilitation. WS prevalence in the Pacific Ocean has been linked with high densities of hard coral cover [13] and, although not examined in their study, this may include high densities of host cover. When *Acropora* cover is specifically examined it is found to positively correlate with WS outbreaks [15]. If host density of susceptible corals increases disease prevalence, then disease outbreaks may limit the future abundance and distribution of susceptible taxa. In general, high abundance and homogenous spatial distribution of the host population facilitates disease outbreaks, but outbreaks cannot persist once a host population exceeds a threshold level of spatial heterogeneity [39].

WS is among the most destructive coral diseases and this study demonstrates that WS represents a selective pressure that changes the coral community by significantly reducing the abundance of *Acropora* plate corals. These results highlight the serious threat WS poses to future coral reefs given that WS is increasing in distribution and prevalence throughout the Indo-Pacific [2]. The outbreak of WS at Christmas Island adds support to the role of high host cover in WS outbreaks. Determining the proximal cause(s) for WS outbreaks is urgently required for the development of management actions that prevent or mitigate the expected increase in WS.
